# Influence of newborn health messages on care-seeking practices and community health behaviors among participants in the Zambia Chlorhexidine Application Trial

**DOI:** 10.1371/journal.pone.0198176

**Published:** 2018-06-14

**Authors:** Kasthuri Sivalogan, Katherine E. A. Semrau, Paul G. Ashigbie, Sheila Mwangi, Julie M. Herlihy, Kojo Yeboah-Antwi, Bowen Banda, Caroline Grogan, Godfrey Biemba, Davidson H. Hamer

**Affiliations:** 1 Department of Global Health, Boston University School of Public Health, Boston, Massachusetts, United States of America; 2 Emory Global Health Institute at Emory University, Atlanta, GA, United States of America; 3 Ariadne Labs, Harvard T.H Chan School of Public Health, Brigham and Women’s Hospital, Boston, Massachusetts, United States of America; 4 Harvard Medical School, Department of Medicine, Boston, MA, United States of America; 5 Brigham and Women’s Hospital, Division of Global Health Equity, Boston, MA, United States of America; 6 Department of Pediatrics, School of Medicine, University of California Davis, Davis, California, United States of America; 7 Zambian Center for Applied Health Research and Development Limited, Lusaka, Zambia; 8 Section of Infectious Diseases, Department of Medicine, Boston Medical Center, Boston, Massachusetts, United States of America; Johns Hopkins School of Public Health, UNITED STATES

## Abstract

**Background:**

Identifying and understanding traditional perceptions that influence newborn care practices and care-seeking behavior are crucial to developing sustainable interventions to improve neonatal health. The Zambia Chlorhexidine Application Trial (ZamCAT), a large-scale cluster randomized trial, assessed the impact of 4% chlorhexidine on neonatal mortality and omphalitis in Southern Province, Zambia. The main purpose of this post-ZamCAT qualitative study was to understand the impact of newborn care health messages on care-seeking behavior for neonates and the acceptability, knowledge, and attitudes towards chlorhexidine cord care among community members and health workers in Southern Province.

**Methods & findings:**

Five focus group discussions and twenty-six in-depth interviews were conducted with mothers and health workers from ten health centers (5 rural and 5 peri-urban/urban). Community perceptions and local realities were identified as fundamental to care-seeking decisions and influenced individual participation in particular health-seeking behaviors. ZamCAT field monitors (data collectors) disseminated health messages at the time of recruitment at the health center and during subsequent home visits. Mothers noted that ZamCAT field monitors were effective in providing lessons and education on newborn care practices and participating mothers were able to share these messages with others in their communities. Although the study found no effect of chlorhexidine cord washes on neonatal mortality, community members had positive views towards chlorhexidine as they perceived that it reduced umbilical cord infections and was a beneficial alternative to traditional cord applications.

**Conclusion:**

The acceptability of health initiatives, such as chlorhexidine cord application, in community settings, is dependent on community education, understanding, and engagement. Community-based approaches, such as using community-based cadres of health workers to strengthen referrals, are an acceptable and potentially effective strategy to improve care-seeking behaviors and practices.

## Introduction

Neonatal mortality accounts for 2.6 million deaths annually, approximately 46% of all under-five deaths worldwide, and more than 80% of these deaths are preventable [1]. Preterm birth complications, intrapartum-related complications, sepsis and meningitis are leading causes of global neonatal mortality [2]. In 2013, the Zambian neonatal mortality rate (NMR), at 24 deaths per 1,000 live births, was twice the target rate defined by Sustainable Development Goal Three at 12 deaths per 1,000 live births [3]. While postnatal, infant, and child mortality rates have substantially decreased, the NMR in Zambia remains unacceptably high and steps must be taken to reduce preventable neonatal deaths [3]. Identifying newborn care practices and traditional experiences that influence care-seeking behavior in Zambia is crucial to implementing evidence-based sustainable interventions to reduce neonatal mortality.

To address this challenge, the Zambia Chlorhexidine Application Trial (ZamCAT), a community-based cluster randomized controlled effectiveness trial, compared the impact of daily cord cleansing with 4% chlorhexidine against the standard practice of dry cord care on neonatal mortality. The main trial was conducted from February 2011 to October 2013 in Southern Province, Zambia [4] and a baseline qualitative study and intensive community engagement were conducted in Southern Province [5,6]. The results from the baseline qualitative study aimed to identify local perceptions of umbilical cord health, illness, and cultural perception that shape cord care knowledge, attitudes and practices [5]. This baseline study demonstrated that diverse umbilical cord care practices existed and, at the time of the study, the WHO recommendations of dry cord care were not widely practiced in Southern Province [5]. Community engagement utilized these formative research results to inform possible strategies that minimized participant withdrawal and achieved low loss to follow-up [6].

The main purpose of this post-ZamCAT qualitative study was to understand the impact of newborn health messages on care-seeking behaviors and the acceptability, knowledge, and attitude of ZamCAT participants towards use of chlorhexidine. The four objectives addressed by this analysis were to: 1) determine the current knowledge, attitudes, perceptions, and practices of mothers and health workers regarding postnatal and neonatal care; 2) determine knowledge, attitudes, perceptions, and practices of mothers and health workers regarding neonatal umbilical cord care practices after ZamCAT participation; 3) determine the perception and attitudes of mothers and health workers regarding chlorhexidine acceptability; and 4) evaluate use of the ZamCAT referral system. The ZamCAT referral system, a voluntary system, was based on the use of a symptom checklist for severe disease to screen and refer pregnant women, post-partum mothers and newborns when indicated during home visits. The results of this study are conceptualized using Kleinman’s behavioral framework and model of the ‘Local Health Care System’ [7].

## Methods

### Ethics statement

The University of Zambia Research Ethics Committee (Protocol # 001-01-10) and Boston University Institutional Review Board (Protocol #H-32548) approved the protocols and informed consent forms. A bilingual speaker translated the consent forms, FGDs and IDIs between English and the local language, ChiTonga. The interview moderators discussed the confidentiality of all information provided by the participants and obtained written consent from ZamCAT participants.

### Study site

The study was conducted in Southern Province, Zambia. Zambia is a landlocked African country of 752,000 km^2^ with 65% of Zambia’s 13.1 million inhabitants living in rural areas, and over 80% living on less than $1 USD per day [3]. Southern Province, one of ten provinces in Zambia, consists of 11 districts and has a population of 1.3 million individuals. As reported in the 2013 Demographic Health Survey (DHS), the total fertility rate for the three years preceding the 2013 DHS was 5.3 births per woman and the maternal mortality ratio was 591 deaths per 100,000 live-births [3]. The NMR in Southern Province declined by 37.8% from 37 deaths per 1,000 live births in 2007 to 23 deaths per 1,000 live births in 2013–2014 [3]. While nearly 90% of pregnant women have an initial registration at an antenatal clinic (ANC), only 56% meet the WHO-recommended standard of care at the time of the study of 4 or more antenatal visits [4]. Fifty-six percent of pregnant women in Southern Province deliver in health facilities—the third lowest rate among all ten provinces in Zambia. [3]. Forty-two percent of Southern Province newborns did not attend a postnatal checkup within one week after birth, and newborns delivered outside a health facility were less likely to receive a postnatal exam than newborns delivered in a health facility [3].

ZamCAT participants attended between 3.3 (chlorhexidine arm) and 3.4 (dry cord care arm) antenatal visits and facility-based delivery was significantly higher for ZamCAT study participants, at 63.8%, compared to facility-based delivery rates in the Southern Province [4]. During the course of the ZamCAT study, field monitors conducted home visits and provided Zambian Ministry of Health approved newborn care messages (Table 1) to help mothers understand how best to provide care for their babies.

**Table 1:** ZamCAT newborn health messages.

Do not bathe for at least 6 hours after birth. Only bathe her/him when necessary, and after bathing, thoroughly dry your baby and then dress and keep her/him warm
Keep cord stump loosely covered with a clean cloth. Fold diaper and clothes below stump
Do not put anything on the stump (dry cord care group only) OR daily chlorhexidine washing with emphases on importance of early and daily wash (chlorhexidine group only)
If stump area is soiled, wash with clean water and soap, then dry completely with a clean cloth
Wash your hands with soap and water before and after cord care, including the application of chlorhexidine
Keep your newborn warm
Start breastfeeding within 1 hour of birth
During the first 6 months of life, the baby needs nothing more than breast milk–not water, milk, cereals, teas, or juices
Go to the nearest health facility if you or your newborn develops signs or symptoms of serious illness

### Data collection

Data collection was performed immediately following the end of ZamCAT from November 2013 to January 2014. Health centers in six of the Southern Province districts, in Choma, Kalomo, Livingstone, Mazabuka, Monze, and Siavonga, were randomized to the chlorhexidine study arm or the dry cord care arm. Five focus group discussions (FGD) were conducted with 6–10 mothers per FGD. Two FGDs were conducted with mothers who participated in the chlorhexidine study arm. The remaining three FGDs were conducted with mothers who participated in the dry cord care arm of ZamCAT and, based on their study participation, had minimal or no knowledge of chlorhexidine. Twenty-six in-depth interviews (IDIs) were conducted, across all six study districts, with health workers at ten health centers—five in rural sites and five in urban/peri-urban sites. The main trial randomized at the facility level; thus, districts had both intervention and control sites. In this study, nine IDIs were conducted with health workers from chlorhexidine study arm sites, eleven IDIs were conducted with health workers in the dry cord care study sites, and six IDIs were conducted with health workers in District Medical Offices. Health workers who participated in the IDIs included health center staff, nurses, midwives, environmental health technicians (EHTs), and district maternal child health coordinators.

In the main trial, ZamCAT field monitors, who were female community members hired for the trial to complete data collection via home visits, conducted one ANC home visit and four postnatal home visits. From this study population, the ZamCAT field monitors identified and recruited participants. Mothers were eligible if they and their newborns participated in ZamCAT within the last three months and if they were willing to provide written informed consent. The field monitors contacted potential participants, explained the study purpose and proposed a meeting date, time, and place if the individual agreed to participate. On the day of the interview, the field monitors obtained consent from the participant(s) in the participants’ local language and informed the participant(s) that they would not be compensated for participation, were not obligated to participate, and could refuse to answer any question they do not want to answer.

The FGDs and IDIs were conducted in quiet places in the community and each lasted between 60–90 minutes, using a semi-structured discussion guide with an interviewer, note-taker, and the participant(s) present. The study team reviewed each audio recording and written records from each session at the end of each day. The interviewers and note-takers were former midwives who had previous experience collecting qualitative data.

There were three main categories discussed in the FGDs and IDIs: general neonatal care and delivery practices, umbilical cord care in neonates, and chlorhexidine acceptability. For the last set of questions, mothers in the FGDs were asked about chlorhexidine use practices during ZamCAT and health workers in the IDIs were asked about newborn care policy and strategies. Mothers in the chlorhexidine arm were asked about how, when, and how often chlorhexidine was applied. All mothers were asked a variety of post-natal practices including newborn drying, bathing, topic application, feeding, clinical visits and seeking medical care.

### Data management and analysis

The handwritten transcripts were translated into English by the interviewer and entered into NVivo data analysis software (Version 11.1, QSR International, Cambridge, MA). Each of the FGD transcripts and all of the IDI transcripts were coded by two research assistants (KS, SM). Key themes were identified by both research assistants and reconciled during group review with the principal investigator (DHH). A grounded theory approach was used for the data analysis; research assistants assigned codes based on themes and categories of data observed [8]. The IDI data were analyzed with a focus on individual viewpoints and the proportion of total respondents who reported a similar viewpoint. The proportion of respondents who reported similar viewpoints was examined among the FGDs while paying attention to specific outliers or the minority of respondents who expressed viewpoints different from the majority. From the analyzed data, themes on the decision of location of delivery, care-seeking behaviors, factors and barriers that influence care-seeking behavior, including traditions, barriers, and perceptions of ZamCAT neonatal care messages, were identified.

## Results

The FGDs conducted in Monze included 10 Tonga mothers ranging from 19–37 years of age with a median age of 29 years. Demographic data were not available for participants in FGDs that took place in Choma, Kalomo, Livingstone, and Siavonga, due to lost data. Of the IDI participants, nine were enrolled midwives, six were enrolled nurses, two were EHTs, one was a MCH coordinator, one was a clinical officer in charge, one was a classified daily employee, one was a clinical care specialist, one was a lab technologist, one was a district nursing officer, and one was a community health worker (CHW); two IDI participants did not provide position titles.

### Theme I: Care-seeking practices & behaviors

Parents sought care for their newborn to prevent illness, to help monitor the growth of their babies, or when the baby was sick. As indicated in the FGDs, parents’ interpretation of their baby's condition influenced where they chose to seek care. Many participants believed that visiting traditional healers and medicines is appropriate care-seeking behavior for specific ailments. It was common for parents to seek traditional medicines if they found no improvement after taking the baby to the health facility. Statements on care seeking behavior perceived by mothers and health care workers are illustrated in Table 2. It is clear that health workers’ perception of where parents commonly seek medical care is different from the mothers’. Many of the health workers noted that while they advised parents to bring their children to the health facility, they could not supervise or guarantee appropriate health-seeking behavior. Health workers believed that the decision on where to seek care could be made by different individuals including the mother, father or grandmother. Health workers gave varied responses as to where parents would seek care and made references to traditional medicine and community understanding that influenced the decision to seek care and when to seek care.

**Table 2:** Illustrative statements on the perception of parents’ care-seeking behavior.

	According to Parents	According to Healthcare Worker
Perception of Parents’ Care-Seeking Behavior	“To the clinic because that is where medicines are available. We do so because traditional medicines are not recommended to babies” (FGD, Siavonga District)	“I can assure you before they come to the hospital they would have exhausted all these others places, they trust their traditional people, they trust more their TBAs, they trust their “ng’angas”, (witch doctor), to say they will do a better job, first of all they will go, they exhaust, when they see that nothing is happening, they will say “let’s go to the hospital” (Registered Midwife, Choma District)

Common barriers to seeking care, as perceived by mothers, were lack of money for transportation, concerns about availability of staff and medicines at the health facility and disagreements at the home on when or where to seek care. Similar barriers to seeking care were noted by health workers, but barriers such as geography (difficult terrain) and attitudes of the parents were also included. Health workers believed that negative attitudes of parents influenced by prior poor experience with health facilities, fear and/or ignorance about the quality of care at the facility, and lack of health knowledge served as barriers and deterrents to seeking care. One health worker noted that sharing of community members’ personal experiences at the health clinic such as long wait time, insufficient staff, or inadequate supplies were factors that contributed to patients’ skepticism to seek care from the health facility.

According to health workers there were a variety of “care-providers” in the community including traditional healers, *ng’angas*, traditional birth attendants (TBAs), and older women. In Southern Province, traditional healers encompass all individuals who practice non-Western medicine (including herbalists) in the community while *ng’angas* are herbalists that use a combination of herbal medicine and spiritual or divine inspirational healing. The motivating factor for seeking traditional care, among ZamCAT participants, was trust in traditional sources of healing. Many health workers reported that care at the health facility is chosen by parents as a last resort when all else fails or the baby remains unwell. From discussions with the mothers, it was unanimously clear that the mother or both parents made the decision about when and where to seek care for their child.

There were many traditional understandings involving newborn care that influenced care-seeking practices in Southern Province. In Zambia, the Ministry of Health recommends that babies have postnatal care visits at 6–7 days and 6 weeks after delivery [10]. Many health workers noted that it was a challenge to have a postnatal care visit 6–7 days after delivery. Many parents do not want their small babies to be exposed to the environment shortly after birth and thus would only chose to attend the six-week postnatal care visit.

### Theme II: Influence of ZamCAT on care-seeking practices & behaviors

ZamCAT mothers were asked about the education they received from the field monitors, their perceptions of ZamCAT newborn care messages, and their clinic attendance. Mothers agreed that they learned about newborn care practices from the field monitors including how to appropriately clean the umbilical cord, to avoid applying substances to the cord, and to exclusively breastfeed for six months.

The ZamCAT mothers were very clear that the lessons and education they received improved care of their newborns. Mothers revealed that they shared these lessons with mothers who did not participate in ZamCAT, thus allowing other mothers to become knowledgeable on newborn care practices. One recommendation that was reiterated was the need to provide lessons to all village members, including husbands and community leaders, so that all are knowledgeable on newborn care practices. Mothers suggested that improving the messages and distributing them through media campaigns and community meetings would increase understanding of the importance of newborn care practices and encourage mothers to seek antenatal (ANC) and postnatal care from the facility. The desire for education and to gain knowledge on ANC, newborn and postnatal care practices were principal reasons for mothers’ attendance at the clinic.

Health workers commented on both positive and negative perceptions of ZamCAT. One positive component of ZamCAT was the use of field monitors, as they were local community members, and helped address and dissuade potentially negative traditional perceptions on caring for the newborn and preventing umbilical cord infections. ZamCAT mothers discussed how the community messages modified their newborn care practices and as a result, health workers claimed to witness fewer cases of infected umbilical cords. Health workers had positive perceptions of ZamCAT as participating mothers were given crucial information and communicated this information to non-ZamCAT mothers. As understood by the health workers, this sharing of information allowed mothers, including those who did not participate in ZamCAT, to identify danger signs and seek the necessary care to ensure that their children were properly cared for.

The only negative perceptions described by health workers were in response to individual perceptions of ZamCAT and the educational content. Health workers described a few negative perceptions of ZamCAT in the context of individuals’ perceptions of ZamCAT including the educational content given to the individuals. Some community members initially believed that ZamCAT was associated with satanic practices and rejected the newborn care and umbilical cord care messages. In the IDIs, health workers noted that elderly women in the community chose to reject ZamCAT because these women had previously delivered children without ANC, chlorhexidine, or postnatal care.

There were mixed impressions of the impact of ZamCAT messages on both care-seeking practices and routine clinic attendance. Health workers noted that the positive impact of the ZamCAT educational messages and the field monitors’ encouragement for mothers to seek care and treatment from the facility were reasons for increased clinic attendance. One rationale for poor clinic attendance was because mothers believed that they had knowledge on newborn care, were able to manage their newborn’s health and only chose to seek care from the facility for complications. Both positive and negative impressions of the influence of ZamCAT on care seeing behavior are presented in Table 3.

**Table 3:** Illustrative statements on care-seeking practices & behaviors and influence of ZamCAT.

	Illustrative Statements on Care-Seeking Practices & Behaviors	Illustrative Statements on the Influence of ZamCAT on Care-Seeking Practices & Behaviors
Focus Group Discussions	“Messages were very effective and well received by the community. It is just that some don’t practice them” (FGD, Livingstone District)	“Was good because after they finished teaching us, we also went to teach others in the community and they also became knowledgeable and came in numbers to antenatal clinic” (FGD, Monze District)
In-Depth Interview	“It is just the attitude of the parents. Others would think the condition will change and get back to normal so they will wait a bit, others may decide to go to the traditional healers” (Registered Midwife, Kalomo District) “The same [fear of] distance, fear of stigmatization, and fear of exposing the baby to other babies. What does one do or what would a caregiver do if the skin surrounding the baby’s umbilical cord is red? (Clinical Care Specialist, Livingstone District) “The traditional barrier [to seeking care at the facility] is that the baby is too small to move out of home, so the facility will still keep it in the home even if it is sick if they think the baby is too small to be taken out of the home as per traditional belief that it cannot come out until one to three months. Their fears are that if the baby is taken out of the home when too young, then it may catch another disease.” (Enrolled Nurse, Monze)	“The ZamCAT messages affected us in a positive way, meaning that they were very helpful. When the field monitors visited the mother, they were able to detect those danger signs that the mother wouldn’t detect on her own and that was helpful as it made us give treatment to the babies much earlier before something else worse happened. As I said the referral system was good because it made us attend to the children earlier and on time” (Enrolled Nurse, Monze District) “Positively yes. There would be women who could not or [would not] want to bring the baby to the clinic despite seeing the baby with a problem and these field monitors would refer the same baby to the clinic and the baby was attended to” (Zambia Enrolled Midwife, Mazabuka District) “At first it was difficult because you know this program dealing in umbilical cord, at first people thought these people are Satanists, others were even advising those pregnant women not to enroll with the program but afterwards they saw that this program had no Satanism attached, they saw the benefits of it so more started joining” (Registered Midwife, Kalomo District) “ZamCAT mothers we saw very few because they were able to manage the cords of the babies we saw more of those who are non-ZamCAT” (Clinical Care Specialist, Livingstone District)

### Theme III: Chlorhexidine acceptability

Mothers in the chlorhexidine arm of the study were asked about their perception regarding applying chlorhexidine within the first 24 hours of life. While all responders had positive feelings, the reasons behind their feelings varied and, in some cases, contained misconceptions. In Kalomo, mothers had positive perceptions of chlorhexidine because they believed that it helped reduce abnormal smells and infections of the cord. Mothers from Monze were impressed that chlorhexidine would help the baby’s cord to drop faster; however, study data support a longer time to cord drop when chlorhexidine is applied [9]. Mothers from Siavonga believed that chlorhexidine application would be acceptable if health workers would provide education for the community on the application and benefits and if health staff and/or community-based agents (i.e. TBAs) could provide community sensitization. ZamCAT mothers from Kalomo and Monze, both agreed that chlorhexidine should replace traditional applications and specifically should increase chlorhexidine use so that traditional practices can be discontinued for good. Statements regarding positive perception of chlorhexidine acceptability by mothers are illustrated in Table 4.

**Table 4:** Illustrative statements on chlorhexidine acceptability in ZamCAT.

	According to Parents	According to Healthcare Worker
Perception of Chlorhexidine Acceptability	“What impressed me is because that medicine they gave us, so that we could put on the cord of the baby, they used to tell us that this medicine, you should use it on the cord, and I found that it helped my baby’s cord to drop fast” (FGD, Monze District). It would be accepted, as long as we are told, and they explain it benefits and we understand the explanations. As long as there can be some workshops for health staff including TBAs to sensitize their communities” (FGD, Siavonga District).	“According to me the Chlorhexidine actually reduces the chances of infection because the active parts in the Chlorhexidine are very much effective against the germs that cause infection” (Zambia Enrolled Nurse, Kalomo District). “Chlorhexidine should be applied to all newly born babies and not just to specific group of babies” (Community Health Worker, Monze District).

Mothers in the dry cord arm were asked about their perceptions of chlorhexidine despite not using chlorhexidine during the study. Two mothers in Siavonga did not have positive perceptions of chlorhexidine and were concerned that mothers’ lack of knowledge on chlorhexidine would stymie acceptability among the family. One Mazabuka mother compared her perception of chlorhexidine to the traditional fears surrounding HIV, as rumors had continued to persist in the community after mothers believed they were prescribed medicines that would infect their children with HIV. Mothers in Monze were concerned that chlorhexidine would delay time for the umbilical cord to drop or negatively impact their child’s health and two mothers in Kalomo claimed that their babies cord began swelling after chlorhexidine was applied. Mothers in the dry cord care arm reported that they were not familiar with and had no knowledge of chlorhexidine. These responses indicated that the study design was effective and there was no evidence of cross-contamination between the chlorhexidine and dry cord clusters.

Health workers believed that chlorhexidine did not cause harm, was beneficial in preventing infection of the umbilical cord, and was easy to use by mothers to apply on their newborn. Health workers were impressed with the use of chlorhexidine messages to enhance knowledge managing the umbilical cord and the reception of the messages by the community. Health workers similarly agreed with mothers that all users (health workers, mothers, other caretakers, etc.) should receive proper training to dissuade misconceptions and ensure proper use and applicatin for preventing umbilical cord infections. Statements regarding positive perception of chlorhexidine acceptability by healthcare workers are illustrated in Table 4. A health worker in Livingstone compared the need for general chlorhexidine application to the general use of tetracycline, when available, to prevent eye infections. Half of interviewed health workers considered use of chlorhexidine to be unacceptable when there was an indication of an umbilical cord or stump infection. Health workers from Monze believed that chlorhexidine applications should be suspended when a baby shows signs of infection, namely redness, pus or discharge as “it would defy all logic that the cord is getting infected when they are applying chlorhexidine.” As stated by a Kalomo health worker, most health workers believe that “prevention is better than [needing to] cure” and therefore chlorhexidine should be applied to all newborns’ cords. The use of chlorhexidine was positively perceived, as an alternative to traditional cord applications, to reduce umbilical cord infections despite study results that indicated that chlorhexidine did not reduce NMR when compared to dry cord care in Southern Province [5].

A few misconceptions about chlorhexidine surfaced in discussions with mothers and health workers. One mother in Kalomo believed that chlorhexidine use was unacceptable and should not be applied if the umbilical cord was not dropping fast enough. Misconceptions by health workers included that chlorhexidine was an antibiotic or that chlorhexidine would immediately heal the cord and any infections if applied within 24 hours.

### Theme IV: Use of the ZamCAT referral system

During the post-qualitative study, health care workers gave their perspective on the effect of the ZamCAT referral system, community perceptions of ZamCAT messages, clinic attendance, and their recommendations for both ZamCAT and the ZamCAT referral system. Perceptions of the effect and recommendations for the ZamCAT referral system, as noted by healthcare workers, are illustrated in Table 5 below.

**Table 5:** Illustrative statements on use of ZamCAT referral system.

	According to Health Workers
Effect of ZamCAT Referral System	“The ZamCAT messages affected us in a positive way, meaning that they were very helpful. When the field monitors visited the mother, they were able to detect those danger signs that the mother wouldn’t detect on their own and that was helpful as it made us to give treatment to the babies much earlier before something else worse happened. As I said the referral system was good because it made us attend to the children earlier and on time” (Enrolled Nurse, Monze District). “If there was a way of adopting it, it would be very beneficial because the system would continue and these children would be referred just the way they were referred on their own being brought to the clinic as early as possible (RM, Livingstone District)”
Recommendations for ZamCAT Referral System	“The referral system … I would say somehow we had no proper coordination of the same referral system because it like they were doing it on their own, thought we could see people coming but they were doing it on there own” (Enrolled Nurse, Kalomo District). The negative effects were that some mothers that were referred by the field monitors never used to come to the clinic due to unknown reasons (Community Health Worker, Monze District) The negatives that we had was sometimes we would refer a baby to the hospital then the mother on the way will decide when she reaches the hospital she decide to abscond and take the baby maybe to a traditional healer and then eventually you will hear the baby passed away” (Unknown, Livingstone District).

The ZamCAT system for referral of sick mothers and babies was perceived to increase access to the health facility. Mothers were referred to health facilities and field monitors were able to facilitate interactions between mothers and the staff at facilities. As noted by many health workers, this system was effective because health workers were able to conduct home visits which allowed for timely detection of signs and symptoms that may have gone unnoticed by the parents.

The health workers noted two concerns with the ZamCAT referral system. Some health workers perceived that mothers were unnecessarily referred through the system to health centers. It was also noted that some mothers who were in fact referred to the health center did not go to the clinic and therefore did not receive the necessary care for themselves or their infants. Based on these concerns, recommendations to improve this system included providing transportation to facilitate referrals and greater communication with the health center to ensure that mothers received necessary care. Health workers also recommended increasing CHW presence, as field monitors were unique to ZamCAT, to sustain the ZamCAT referral system and increase screening and referral coverage in the communities.

## Discussion

Our findings indicated that community perceptions and local understandings are fundamental to the decision to seek care and influence acceptance of beneficial health-seeking behaviors. From discussions with ZamCAT mothers and health care workers, it is evident that existing traditional perceptions have major influences on care-seeking behavior and practices in the community. Community members were accepting of ZamCAT and believed that community engagement and education on health-seeking behavior associated with newborn health behaviors were beneficial for the participating communities. With the exception of differences in umbilical cord care, there were no major differences in answers by respondents in the six Southern Province districts. Per the 2010 census, the Southern Province is relatively homogenous (74.4% of the Southern Province population is Tonga); thus, we did not expect or observe major difference across the districts [10].

**Fig 1:**
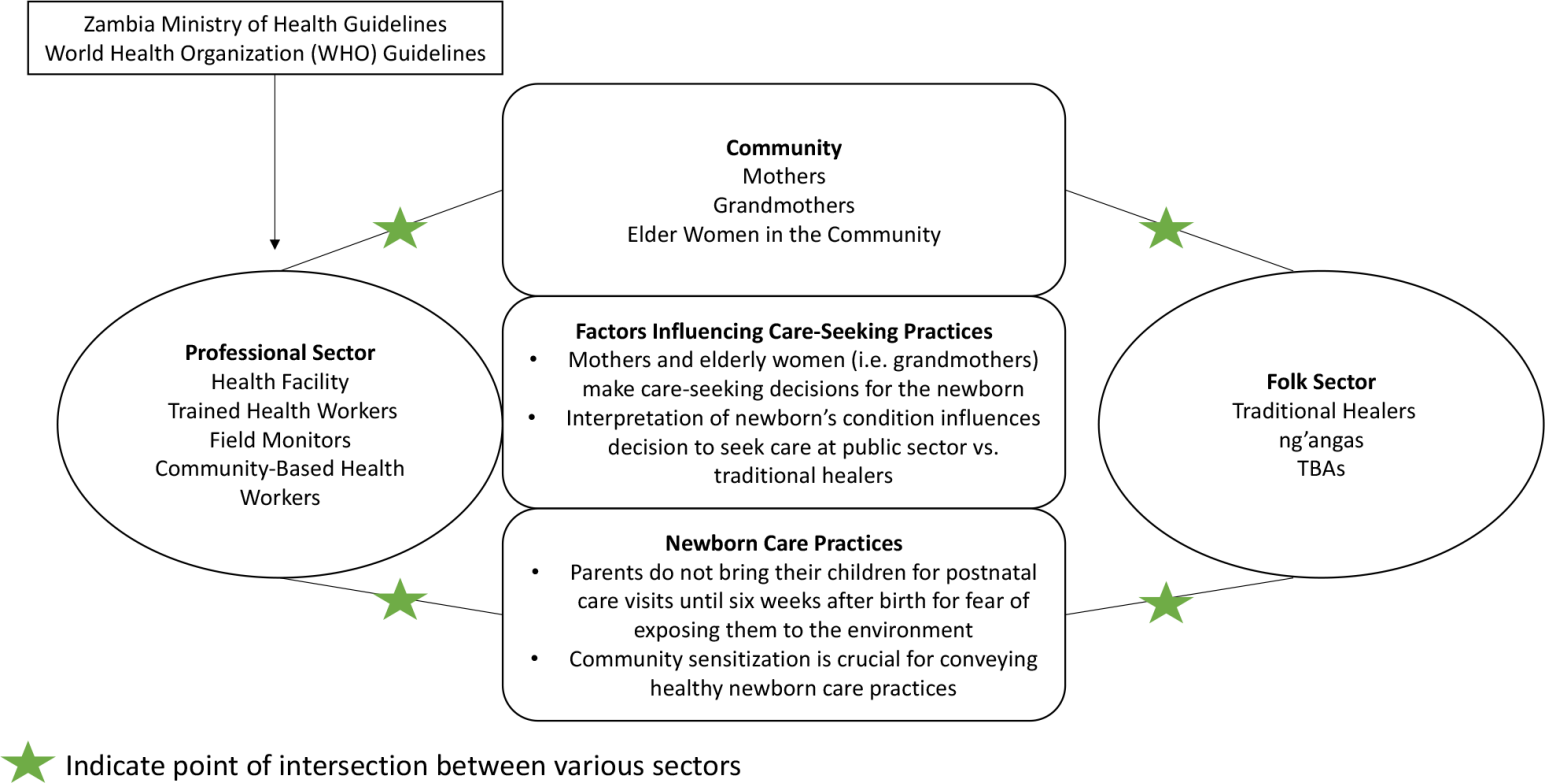
Adaptation of Kleinman’s local health system model as a behavioral model in Southern Province, Zambia.

Kleinman’s model of the “Local Health Care System” is an appropriate behavioral framework to conceptualize the results of this qualitative study [7]. In this model, a society’s health care system is a cultural system that explains how a community copes and responds to illness and includes a folk sector, professional sector and popular sector [7]. All three sectors overlap and are interconnected but represent a particular way of explaining and treating illnesses and understanding care-seeking and newborn care practices in the Southern Province [6]. The folk sector represents the traditional beliefs held by families regarding methods to prevent cord infections and treating newborns and involving care from traditional healers, *ng’angas* and TBAs. The professional sector includes beliefs held by trained health workers and beliefs supported by government policy and modern Western scientific medicine. The popular sector is the largest part of the local health care system and is where lay people and non-professionals–particularly the mothers, grandmothers and older women in communities recognize and make health care decisions. As demonstrated in the adaptation of Kleinman’s model for the Southern Province in Fig 1, it is crucial to address all three sectors when making and delivering effective policies to address care-seeking and newborn care practices. (Fig 1).

Traditional perceptions have a major influence on care-seeking behavior during the postpartum period, particularly in seeking care for newborns. There were several discrepancies between the responses of ZamCAT mothers and health care workers regarding care seeking behavior in the Southern Province. While mothers responded that they would first seek care at the health facility, health care providers perceived that mothers would only come to the health facility if traditional healers were deemed unsuccessful. It was clear from health care provider responses that they believed that mothers and parents relied on traditional perceptions to influence care-seeking decisions, including where and when to seek care.

The influence of traditional perceptions on care seeking behavior was most evident for seeking postnatal care at health facilities. The WHO 2013 guidelines and recommendations for postnatal care for mothers and newborns stated that every mother and baby should be provided with 4 postnatal visits within the first 6 weeks following delivery, regardless of delivery location [11]. If a delivery occurs in the facility, mother and baby should receive postnatal care in the facility for at least 24 hours after birth and the first postnatal contact for home deliveries should occur within the first 24 hours after birth [11]. As described in our study, it is customary for the newborn to remain in the house for a certain period of time, ranging from 2 weeks to 3 months, following birth. In seeking neonatal care within the first month of life, it is apparent that some local understandings serve as a barrier to postpartum care-seeking behavior necessary to ensure newborn health [11]. These same understandings also influenced individuals’ decision to rely on community TBAs when seeking care. TBAs shared the same traditional perceptions and instill a sense of trust because they practice methods that are culturally accepted by the community [12,13]. While this qualitative study did not include participation from TBAs, these individuals play an important role in the community and are an important conduit for communicating appropriate care-seeking behaviors.

The ZamCAT program was well received among all six districts in the Zambian Southern Province. Mothers noted that ZamCAT was effective in providing lessons and education to mothers on newborn care practices and participating mothers were able to share these messages with others in their community. One recurring recommendation was to increase access to newborn health messages for pregnant women, family members, and community members (such as community and religious leaders). Suggestions for improving the distribution of key lessons in the community included having CHWs, Safe Motherhood Action Groups (SMAGs) and trained community volunteers to disseminate information on best practices, provide mothers with access to skilled birth attendants at health facilities and improve and deliver these messages through media campaigns and community meetings.

In the ZamCAT referral system, field monitors conducted home visits and screened pregnant women and their newborns for signs of severe disease and advised treatment at health facilities when positive warning signs were detected. In some circumstances, field monitors were able to contact the health center staff to let them know that a patient was coming but field monitors did not have additional resources to facilitate referrals. Despite the lack of a government-sponsored home visitation and referral program within ZamCAT, many health workers had positive perceptions of the system as they believed that it was effective in identifying signs of severe disease in the newborns. In evaluating the ZamCAT referral system, health workers noted that coordination and sustainability were two main concerns that needed to be addressed for future improvement. Mothers may have unnecessarily been referred to the clinic because the danger sign checklist was based on the WHO’s Integrated Management of Childhood Illness young infant criteria and may have been too sensitive for identifying infants in need of referral [14]. While not all the suggestions are feasible or sustainable, developing an integrated referral system from community-based screening through health center evaluation including feedback to the referring CHWs is necessary. Recruiting CHWs and TBAs to scale up SMAGs would contribute to a more sustainable system in communities. CHWs or TBAs could act as field monitors to educate mothers, screen for severe diseases and communicate with health centers to facilitate referrals. As a part of the national integrated CHW case management program, CHWs currently conduct home visits during the neonatal period and refer sick newborns to the health facility in Eastern Province; this strategy will eventually be implemented in Southern Province [15].

Proper dissemination of results from this study at the community level is necessary for understanding the appropriate role for chlorhexidine cord care. There were some misperceptions in the ZamCAT study communities regarding chlorhexidine, with one misperception being that health workers did not consider chlorhexidine to be harmful. However, it was reported by the Nigerian government that chlorhexidine drops were mistakenly used as eye drops and blinded five children in the Yobe state of Nairobi [16]. While there are no reports of chlorhexidine being considered harmful when used appropriately to treat umbilical cord infections, it is important to correct the misperception that there are no risks associated with chlorhexidine use. A controlled managed access program was implemented in Bungoma County, Kenya to evaluate the acceptability of 7.1% chlorhexidine gel for neonatal umbilical cord care among providers and mothers of newborns [17]. Health care providers accepted use of CHX gel and were largely influenced by positive outcomes including ease of use, fast healing of the cord, minimal side effects and reduced infections based on records and mothers’ reports [16]. The evaluation presented similar results to ZamCAT as mothers and health workers agreed that chlorhexidine should continue to be freely provided through government health facilities and the importance of education and training for mothers and health providers prior to use [17].

There are several strengths and potential limitations to our study that should be noted in examining the impact of this qualitative study. As previously mentioned, mothers were recruited for focus groups from six districts within the Southern Province of Zambia and IDIs were conducted with health workers from ten health centers (5 rural and 5 peri-urban/urban). One strength of the study was that it included diverse participants from recent mothers to health care practitioners and represented a variety of perspectives on newborn care within the Southern Province of Zambia. One limitation is that demographic data were missing for five FGDs, however we were able to describe demographic characteristics for the FGD in Monze. Another limitation is that many of the FGD and IDI participants discussed traditional perceptions or practices that they perceived to exist or had heard that individuals in the community practiced, However, the quantification of individuals who actually shared the discussed understanding remains unclear. Despite these limitations, this post ZamCAT qualitative study has provided fruitful insight into the perceptions that impact health-seeking behaviors among ZamCAT participants. The acceptability of health initiatives, such as chlorhexidine application, in community settings is dependent on community education, understanding, and sensitization. Community-based approaches, such as using CHWs to strengthen referrals, are an acceptable and potentially effective strategy for improving care-seeking behavior and practices.
